# Abus médicamenteux et céphalées chroniques à Brazzaville: profil et parcours thérapeutique des patients

**DOI:** 10.11604/pamj.2019.33.203.7309

**Published:** 2019-07-15

**Authors:** Paul Macaire Ossou-Nguiet, Dieudonné Gnonlonfoun, Karen Lise Obondzo-Aloba, Komi Assogba, Edgard Matali, Dominique Nguiegna, Franck Ladys Banzouzi, Julien Arzur

**Affiliations:** 1 Département de Médecine, Faculté des Sciences de la Santé de Brazzaville, Congo; 2 Service de Neurologie, CHU de Brazzaville, Congo; 3 Département de Neurologie, Centre National Hospitalier Universitaire Hubert Koutoukou Maga, Cotonou, Bénin; 4 Service de Neurologie CHU-Campus, Lomé, Togo; 5 Service de Neurologie, Centre Hospitalier de Lisieux, France

**Keywords:** Abus médicamenteux, céphalées, Brazzaville, Medication-overuse, headaches, Brazzaville

## Abstract

**Introduction:**

Les céphalées par abus médicamenteux (CAM) restent le type de céphalées le moins étudié en Afrique.

**Méthodes:**

Dans le but de rapporter l'expérience Brazzavilloise, nous avons mené une étude longitudinale durant 4 ans, de septembre 2010 à août 2014, en consultation de neurologie à Brazzaville. Nous avons inclus tous les patients présentant des céphalées primaires chroniques selon la *International Classification of Headache Disorders-2* (ICHD-2). Tout patient présentant des céphalées secondaires ou n'ayant pas donné son consentement a été exclu. Les patients ont été divisés en deux groupes: ceux ayant évolué vers une CAM, et ceux qui ne présentent pas des critères d'abus médicamenteux (sans-CAM). Les variables d'études ont été sociodémographiques, les caractéristiques de la céphalée primaire initiale et la prise en charge de la CAM.

**Résultats:**

Sur 212 patients inclus, 193 ont constitué notre population d'étude. L'âge moyen de 42±14 ans, dont 66,32% de femmes. La fréquence des CAM était de 35,75%. Les facteurs associés identifiés étaient: l'âge jeune (p=0,003), l'utilisation de l'association antiinflammatoire non stéroïdien (AINS) et paracétamol (p=0,0001) et l'automédication (p<0,0001). Par contre, le niveau d'instruction supérieur (p<0,0001) et l'utilisation de l'AINS seul (0,002) étaient protecteurs contre la survenue de la CAM. Le sevrage ambulatoire a été le plus pratiqué, l'amitriptyline reste le médicament le plus utilisé.

**Conclusion:**

Les CAM sont fréquentes en consultation de neurologie en Afrique et méritent d'être identifiées pour une meilleure prise en charge.

## Abstract (en)

**Introduction:**

Medication-overuse headaches (MOHs) are the least studied of all headaches in Africa.

**Methods:**

We conducted a longitudinal study in order to describe our experience with patients placed in Neurology Outpatient observation in Brazzaville over a period of 4 years, from September 2010 to August 2014. All patients with chronic primary headaches, according to the International Classification of Headache Disorders (ICHD) (2nd edition), were included in the study. All patient with secondary headaches or who hadn't given consent were excluded. The patients were divided into two groups: those who had progressed to medication-overuse and those who didn't meet the criteria for medication-overuse (without-MOH). The variables used were the sociodemographic characteristics of patients, the clinical features of primary headaches and MOH treatment.

**Results:**

The study population was constituted by 193 patients out of 212. The average age of patients was 42 ± 14 years, of whom 66.32% were women. MOH rate was 35.75%. The associated factors were: early age (p=0.003), concomitant use of nonsteroidal anti-inflammatory drugs (NSAIDs) and paracetamol (p=0.0001) as well as self-medication (p<0.0001). By contrast, higher education (p<0.0001) and the use of NSAID alone (0.002) were protective factors against the onset of MOH. Ambulatory withdrawal was the most practiced treatment and amitriptyline was the most commonly used medication.

**Conclusion:**

Medication-overuse headaches are frequent in patients placed in Neurology Outpatient observation in Africa and deserve identification for a better management.

## Introduction

La version bêta de la 3^ème^ édition de la classification internationale des céphalées (ICHD-3 bêta) [1], définit la céphalée chronique quotidienne par abus médicamenteux comme étant des céphalées présentes au moins 15 jours par mois chez un patient ayant une céphalée préexistante, chez qui on note un abus régulier depuis plus de 3 mois d'un ou de plusieurs médicaments pouvant être utilisés comme traitement des céphalées, et que ces céphalées ne sont pas attribuables à une autre cause. L'abus médicamenteux est défini par le nombre de jours avec consommation d'un traitement de crise, quelle que soit sa quantité journalière, ce nombre étant évalué sur trois mois consécutifs. Cependant, les seuils de prise par mois diffèrent d'une classe médicamenteuse à une autre: au moins 15 jours pour les antalgiques non opioïdes: paracétamol, aspirine, anti-inflammatoires non stéroïdiens (AINS) et au moins 10 jours pour les opioïdes, les triptans, les ergotés et en cas d'association de plusieurs principes actifs ou de plusieurs médicaments [2]. Cette pathologie relativement rare avec une prévalence de 1 à 2% de la population générale [3,4], a cependant un retentissement psychosocial important et son impact sur la qualité de vie est diversement étudié [2,5]. Dans le contexte africain, particulièrement au Congo, aucune étude n'a abordé le profil des patients suivis pour céphalées par abus médicamenteux. Nous rapportant une série brazzavilloise dans le but de décrire le profil des patients et leur parcours thérapeutique.

## Méthodes

Il s'agit d'une étude longitudinale du 02 septembre 2010 au 31 aout 2014, soit 4 ans, réalisée en consultation externe de neurologie au CHU de Brazzaville et en consultation libérale de neurologie. Les critères d'inclusion ont été des patients présentant des céphalées chroniques primaires selon la classification ICHD 2 [6], ayant donné leur consentement éclairé, et leur accord d'être suivi sur le long terme. Pour éliminer toute autre pathologie, tous les patients inclus ont bénéficié d'un scanner cérébral. Les patients inclus étaient divisés en 2 groupes: ceux ayant évolué vers une céphalée par abus médicamenteux (CAM), et ceux qui ne présentent pas des critères ICHD d'abus médicamenteux (sans-CAM). N'ont pas été inclus tous les patients présentant des céphalées secondaires, et ceux ayant refusé de donner leur consentement. Les variables de l'étude ont été l'âge, le sexe, le niveau d'instruction, le type et la durée d'évolution de la céphalée primaire initiale, la fréquence mensuelle des crises durant les 3 mois précédents la surconsommation des médicaments pour le groupe CAM, les médicaments utilisés, la qualification du médecin consulté les 3 derniers mois, les modalités de sevrage et l'évolution. La surconsommation des médicaments avait été étudiée de façon rétrospective, à l'interrogatoire, sans agenda de céphalées primaires pour les patients n'ayant pas eu accès au neurologue avant, et de façon prospective avec agenda de la céphalée primaire pour tous les patients suivis par un neurologue et à l'inclusion dans l'étude. L'analyse statistique a été réalisée par le logiciel Epi info. Les variables quantitatives ont été exprimées en moyenne ± écart type et les variables qualitatives en pourcentage. Le test t de student a été utilisé pour comparer les moyennes et le test de Chi^2^ de Pearson pour comparer les pourcentages. Le modèle de régression linéaire a permis d'identifier les facteurs associés à la surconsommation des médicaments. Seuls les facteurs avec une valeur de p < 0,20 ont été retenus pour une analyse multivariée. Le seuil de significativité statistique a été fixé à p = 0,05.

## Résultats

**Figure 1:**
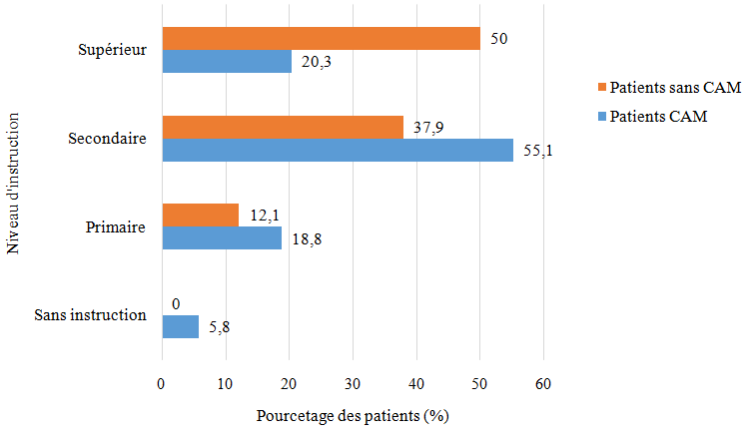
Répartition de la fréquence des patients selon le niveau d'instruction

Durant la période d'étude 212 patients ont été vus pour céphalées primaires, dont 6 n'ont pas donné leur consentement, et 13 ont été perdus de vus après la première consultation. Notre étude a porté donc sur 193 patients. L'âge moyen des patients était de 42 ± 14ans, avec 128 (66,32%) femmes et 65 (33,68%) hommes. Le groupe des patients présentant une CAM était de 69 patients soit 35,75% de l'ensemble des patients. Dans ce groupe, l'âge moyen était de 37 ± 12 ans (extrêmes: 18-67 ans), les femmes représentaient 73,9% (n = 51) et les hommes 26,1% (n = 18). Dans le groupe sans-CAM représenté par 124 (64,25%) dont 77 (62,1%) femmes et 47(37,1%) hommes, l'âge moyen était de 44± 11. La répartition selon le niveau d'instruction des deux groupes est représentée par la Figure 1. Les céphalées primaires étaient représentées par la migraine dans 96 cas (49,7%), la céphalée de tension dans 59 cas (30,6%), 36 cas d'association migraine et céphalée de tension (18,7%) et 2 cas d'algie vasculaire de la face. Aucun des 2 patients présentant une algie vasculaire de la face n'a présenté un abus médicamenteux. Dans le groupe CAM, la durée moyenne d'évolution des céphalées était de 6 ± 2,2 ans chez les migraineux, de 2±1,8 ans dans le groupe des céphalées de tension, de 2±1,2 ans dans le groupe association céphalées de tension et migraine. Le délai moyen de la première consultation d'un neurologue était de 5±1,8 ans chez les migraineux, 2±0,7 ans pour les céphalées de tension et 1±0,4 ans pour l'association migraine et céphalées de tension.

Le Tableau 1 représente l'analyse multivariée des différentes variables dans les deux groupes, identifiant les facteurs associés à l'abus médicamenteux. Les triptans n'ont été prescrits que chez 6 patients migraineux, parmi lesquels aucun n'a présenté une CAM. Concernant les modalités de sevrage dans le groupe CAM, 67 patients (97,1%) ont bénéficié d'un sevrage en ambulatoire associé à une éducation thérapeutique, et 2 patients (2,9%) ont nécessité une hospitalisation de 5 jours. Dans le cadre du sevrage, l'amitriptyline a été utilisé chez tous les patients en per os à la dose de 5 à 25mg, puis jusqu'à 75mg en injectable pour les 2 patients hospitalisés. Chez les patients présentant une céphalée de tension avec abus médicamenteux, 77,3% (n=17) une association avec clonazépam 0,25 à 1mg a été utilisée. Chez les migraineux l'association au clonazépam a concerné 23,3% (n=7), et le valproate de sodium dans 36,7% (n=11). Tous les patients ont été suivis pendant au moins six mois, après le sevrage, avec un retour au caractère épisodique de la céphalée primaire.

**Tableau 1:** Analyse multivariée des facteurs associés à la survenue des céphalées par abus médicamenteux (CAM)

Variables	Patients avec CAM (n=69)	Patients sans CAM (n=124)	OR [IC 95%]	*P*
Age moyen (jeune)	37 ± 12	44 ± 11	1,68 [1,21-3,12]	0,003
Sexe féminin	51(73,9%)	77(62,1%)	1,26 [0,82-2,33]	0,09
**Niveau d’instruction**				
Sans instruction	4(5,8%)	0	/	/
Primaire	13(18,8%)	15(12,1%)	1,32 [0,46-2,58]	0,36
Secondaire	34(49,3%)	47(37,9%)	1,53 [0,65-3,43]	0,42
Supérieur	18(26,1%)	62(50%)	0,31 [0,12-0,73]	<0,0001
**Médicaments utilisés pour la crise**				
Paracétamol seul	11(15,9%)	21(16,9%)	1,04 [0,49-2,11]	0,48
Tramadol seul	0	2(1,6%)	/	
AINS seul	13(18,8%)	38(30,6%)	0,69 [0,57-0,94]	0,002
Corticoïdes*	4(5,8%)	0	/	
Ergotés*	13(18,8%)	15(12,1%)	1,81 [0,69-2,84]	0,67
Paracétamol + Tramadol	6(8,7%)	8(6,5%)	1,54 [0,65-3,45]	0,52
Paracétamol + Codéine	8(11,6%)	21(16,9%)	0,87 [0,52-2,33]	0,12
Paracétamol + Codeine + Cafeïne	5(7,2%)	8(6,5%)	1,43 [0,85-3,03]	0,61
AINS + Paracétamol	26(37,7%)	26(20,2%)	2,11 [1,24-3,16]	0,0001
**Modalité d’acquisition du médicament**				
Automédication	58(84%)	48(38,7%)	4,31 [2,47-8,11]	<0,0001
Prescription médicale	67(97%)	124(100%)	0,92 [0,68-2,01]	0,58
**Qualification du médecin consulté les 3 derniers mois**				
Médecin généraliste ou Autres spécialistes	52(75,4%)	(90,3%)	0,77 [0,39-2,12]	0,64
Neurologue	15(21,7%)	97(78,2%)	0,32 [0,12-0,58]	<0,0001
Aucun	2(02,9%)	0	/	/

* Médicaments pris en association avec les autres médicaments

## Discussion

L'abus médicamenteux est un phénomène fréquent dans les céphalées chroniques, bien que sa prévalence en population générale soit faible dans les pays industrialisés [3,4]. Notre étude, réalisée en consultation spécialisée de neurologie, présente un biais de sélection et ne peut rendre compte de la prévalence des céphalées par abus médicamenteux dans la population brazzavilloise. De même, cette étude présente certaines limites du fait que les facteurs de risque et les comorbidités décrites par Lantéri-Minet *et al.* [2], tels que l'anxiété généralisée, épisodes dépressifs avérés, événements biographiques stressants, douleurs musculo-squelettiques, les troubles ventilatoires du sommeil et excès pondéral n'ont pas été abordés. Cependant en pratique hospitalière et libérale cette étude est la première au Congo. La fréquence des CAM de 35,75% de l'ensemble des patients céphalalgiques chroniques prouve que c'est une pathologie fréquente en milieu africain, cette fréquence est probablement mal-estimée, du fait que certains patients céphalalgiques restent suivis par les médecins généralistes, et que les critères ICHD ne sont suffisamment pas divulgués auprès de ces derniers dans le contexte Congolais.

Davies [7] dans son commentaire rapporte en pratique générale et en neurologie la fréquence des CAM est respectivement de 6 et 9%, mais dans les cliniques spécialisées cette fréquence varie entre 30 et 50% comme l'ont aussi rapporté certains auteurs [8-10], ce qui corrobore nos résultats. Comme nos résultats l'indiquent, s'il est admis que les femmes sont plus sujettes à la migraine, les céphalées de tension sans nécessairement être exposées aux CAM [2,3,11], le jeune âge retrouvé comme facteur associé à la survenue des CAM dans notre étude n'a pas été rapporté. Westergaard *et al.* [11] au Danemark ont rapporté que les CAM étaient beaucoup plus fréquentes dans la tranche d'âge de 35 à 65 ans. L'influence de l'âge sur les CAM, passe probablement par la survenue des évènements stressants dans la vie comme l'ont rapporté certains auteurs [12,13], et qu'au Congo ces évènements sont moins bien vécus chez les sujets jeunes, les conduisant à une surconsommation médicamenteuse. Selon les différents types de céphalées primaires Diener *et al.* [14] rapporte une meta-analyse de 29 études comprenant 2612 patients présentant les CAM, dont 65% étaient migraineux, 27% présentaient des céphalées de tension, et 8% une association des deux types. Nos résultats avec des fréquences respectives de 49,7%, 30,6% et 18,7% pour la migraine, les céphalées de tension et l'association céphalée de tension et migraine corroborent les données de littérature avec légère diminution de la fréquence des migraineux, et une légère augmentation de l'association migraine et céphalée de tension. Cependant dans cette même méta-analyse [14], les durées d'évolution rapportées, des céphalées primaires d'environ 20 ans, celles de l'abus médicamenteux d'environ 5 ans, sont beaucoup plus longues que dans notre série. Ceci s'explique par le fait que nous n'avons pas suffisamment de recul, pour apprécier les récidives et évaluer à long terme les patients actuellement suivis dans la population d'étude. Toutefois, il semble que les patients présentant des céphalées de tension et l'association migraine et céphalées de tension font recours aux médecins, plus tôt que les patients migraineux. La relation entre le niveau d'instruction ou le niveau socioéconomique et la survenue des CAM a été largement rapportée [2,3,11,14]. Notre étude montre de façon intéressante que le niveau d'éducation supérieur est un facteur protecteur. Le médecin généraliste reste le plus accessible et plus consulté à Brazzaville pour un premier épisode de céphalée, du fait que Brazzaville, pour une population estimée à 1.373.382 habitants en 2007, ne compte que 5 neurologues, dont 3 partagent une activité publique-privée. La fréquence élevée et l'intensité des crises restent les 2 facteurs motivant un recours au neurologue dans le contexte congolais, et que la non-accessibilité à un neurologue était associée au risque d'évoluer vers une CAM, ce qui réconforte l'idée de Calabresi et Cupini [15] qui estime que les faibles connaissances des médecins peuvent majorer le risque de CAM.

Sur le plan thérapeutique, comme le montre nos résultats, les triptans sont très peu utilisés en Afrique, même en consultation de neurologie, du fait de leur coût élevé contrastant avec de bons résultats obtenus avec les AINS. Wahab et Ugheoke au Nigéria [16] ont rapporté une enquête de 145 migraineux dont aucun n'avait bénéficié d'un traitement par triptan. L'automédication retrouvée comme facteur de risque des CAM dans notre étude a été estimé à environ 50% des patients céphalalgiques rapporté par l'Atlas de l'Organisation mondiale de la santé en 2011 [17]. L'association régulière AINS et paracétamol apparaît comme un facteur indépendant de risque de CAM dans notre étude, alors que l'utilisation de l'AINS seule apparaît comme un facteur protecteur et le paracétamol seul n'est statistiquement pas associé au CAM. Les données de la littérature notent que tous les médicaments utilisés dans les céphalées peuvent potentiellement occasionner les CAM, cependant les triptans et les AINS bien utilisés chez le migraineux ne majorent pas ce risque [2,14], mais que les relations entre les médicaments et les CAM restent complexes, et ont changé durant ces dernières années [14,18]. Les modalités de prise rapportées dans notre étude sont celles rapportées dans la littérature, le sevrage en antalgique étant l'attitude la plus recommandée [2,3,14]. L'hospitalisation ou non du patient pour le sevrage reste discutée, mais les sevrages en ambulatoire sont actuellement les plus utilisés [2]. L'amitriptyline utilisé dans notre étude était le traitement de référence, mais les données actuelles recommandent le topiramate en première intention [2,3]. Le valproate de sodium est aussi proposé en traitement fond avec une bonne éfficacité au cours du sevrage [14]. Par contre clonazépam utilisé avec une grande efficacité dans le sous-groupe CAM et céphalées de tension, ne ressort pas encore de façon claire dans les recommandations comme une option thérapeutique. Les moyens non pharmacologiques tels que la psychothérapie et l'éducation thérapeutique gardent un rôle clé dans la prise en charge [18].

## Conclusion

La céphalée par abus médicamenteux reste une pathologique fréquente, mais sous-estimée en Afrique subsaharienne. Il s'agit d'une pathologie multifactorielle, on y incrimine, le stress, la mauvaise prise en charge des céphalées primaires telles que la migraine et des céphalées de tension et les facteurs socio-économiques. Sa prévention et sa prise en charge restent accessibles. Les études épidémiologiques méritent d'être réalisées en population générale, afin d'en déterminer les caractéristiques et le retentissement sur la qualité de vie.

### Etat des connaissances actuelles sur le sujet

- La céphalée chronique quotidienne par abus médicamenteux comme étant des céphalées présentes au moins 15 jours par mois chez un patient ayant une céphalée préexistante, chez qui on note un abus régulier depuis plus de 3 mois d'un ou de plusieurs médicaments pouvant être utilisés comme traitement des céphalées, et que ces céphalées ne sont pas attribuables à une autre cause. L'abus médicamenteux est défini par le nombre de jours avec consommation d'un traitement de crise, quelle que soit sa quantité journalière, ce nombre étant évalué sur trois mois consécutifs;
- Les seuils de prise par mois diffèrent d'une classe médicamenteuse à une autre: au moins 15 jours pour les antalgiques non opioïdes: paracétamol, aspirine, anti-inflammatoires non stéroïdiens (AINS) et au moins 10 jours pour les opioïdes, les triptans, les ergotés et en cas d'association de plusieurs principes actifs ou de plusieurs médicaments;
- Cette pathologie relativement rare avec une prévalence de 1 à 2% de la population générale.

### Contribution de notre étude à la connaissance

- Retentissement psychosocial important et son impact sur la qualité de vie;
- Dans le contexte africain, particulièrement au Congo, aucune étude n'a abordé le profil des patients suivis pour céphalées par abus médicamenteux;
- Nous rapportant une série Brazzavilloise dans le but de décrire le profil des patients et leur parcours thérapeutique.

## Conflits d’intérêts

Les auteurs ne déclarent aucun conflit d’intérêts.

## Remerciements

Nos remerciements vont à l' endroit de tout le personnel du service de consultation externe de l'Hôpital Général de Douala et de tous les patients qui ont accepté de participer à cette étude.
